# Operando Study Insights into Lithiation/Delithiation Processes in a Poly(ethylene oxide) Electrolyte of All-Solid-State Lithium Batteries by Grazing-Incidence X-ray Scattering

**DOI:** 10.1021/acsami.4c01661

**Published:** 2024-06-24

**Authors:** Yuxin Liang, Tianle Zheng, Kun Sun, Zhuijun Xu, Tianfu Guan, Fabian A.C. Apfelbeck, Pan Ding, Ian D. Sharp, Yajun Cheng, Matthias Schwartzkopf, Stephan V. Roth, Peter Müller-Buschbaum

**Affiliations:** † TUM School of Natural Sciences, Department of Physics, Chair for Functional Materials, James-Franck-Str. 1, Garching 85748, Germany; ‡ Walter Schottky Institute and Physics Department, Technical University of Munich, Am Coulombwall 4, Garching 85748, Germany; § Ningbo Institute of Materials Technology & Engineering, Chinese Academy of Sciences, 1219 Zhongguan West Rd, Ningbo, Zhejiang Province 315201, P. R. China; ∥ Deutsches Elektronen-Synchrotron DESY, Photon Science, Notkestr. 85, Hamburg 22607, Germany; ⊥ Department of Fibre and Polymer Technology, KTH Royal Institute of Technology, Teknikringen 56-58, Stockholm SE-100 44, Sweden

**Keywords:** all-solid-state lithium batteries, composite electrolyte, poly(ethylene oxide), operando study, X-ray scattering

## Abstract

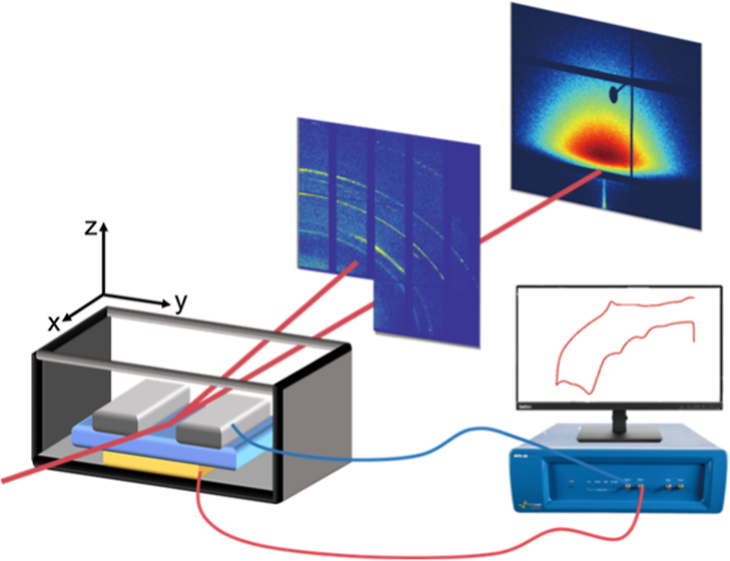

Poly(ethylene oxide) (PEO)-based composite electrolytes (PCEs) are considered as promising candidates for next-generation lithium-metal batteries (LMBs) due to their high safety, easy fabrication, and good electrochemical stability. Here, we utilize *operando* grazing-incidence small-angle and wide-angle X-ray scattering to probe the correlation of electrochemically induced changes and the buried morphology and crystalline structure of the PCE. Results show that the two irreversible reactions, PEO-Li^+^ reduction and TFSI^–^ decomposition, cause changes in the crystalline structure, array orientation, and morphology of the PCE. In addition, the reversible Li plating/stripping process alters the inner morphology, especially the PEO-LiTFSI domain radius and distance between PEO-LiTFSI domains, rather than causing crystalline structure and orientation changes. This work provides a new path to monitor a working battery in real time and to a detailed understanding of the Li^+^ diffusion mechanism, which is essential for developing highly transferable and interface-stable PCE-based LMBs.

## 1 Introduction

Rechargeable lithium-ion batteries (LIBs) are central to the revolution of energy storage technologies and have been successfully commercialized for a wide range of applications, from cell phones to electric vehicles.^1−3^ The low theoretical capacity of the dominant anode material, graphite (372 mA h g^–1^), has spurred the development of lithium-metal batteries (LMBs), in which the anode is replaced with metallic lithium, thereby yielding substantially increased theoretical capacity, reduced density, and low electrode potential.^4−8^ While LMBs with a liquid electrolyte have reached high conductivities and good interfacial performance characteristics, the combustible and toxic electrolyte still suffers from safety hazard and leakage issues.^9,10^ Switching from liquid electrolytes to solid-state electrolytes, such as polymers and ceramics, can not only solve these safety concerns but also facilitate the commercialization of LMBs. In particular, flexible composite polymer electrolytes, consisting of a polymer matrix, lithium salts, and ceramic fillers, provide good mechanical properties and high electrochemical performance and improved interfacial compatibility while also overcoming safety limitations.^11^ Within this class, poly(ethylene oxide) (PEO)-based composite electrolytes (PCEs) are considered among the most promising systems due to their low cost, nontoxicity, and good compatibility with lithium salts.^12^ Within LMBs, the PCE can react with the metallic lithium anode and form a solid electrolyte interface (SEI) layer, while enabling the transport of Li^+^. However, the unstable SEI limits the columbic efficiency (CE) and is ineffective at inhibiting harmful dendrite growth.^13−15^ Despite its importance, the Li plating–stripping process is still not clearly understood and requires further investigation to overcome these hurdles. Indeed, an improved understanding of how the structure and morphology of the PCE evolve during the Li platting/stripping process is of particular significance for the realization of high-performance all-solid-state LMBs via rational design and modification of electrolytes.

Synchrotron radiation-based X-ray probes are among the most effective methods for in situ or *operando* studies of LMBs, offering high luminance and flux, a large range of photon energies, and nondestructive ability. Previous studies showed the possibility of utilizing synchrotron-radiation-based X-ray analytical facilities to gain insights into LIBs. For instance, by using synchrotron-radiation-based X-ray absorption spectroscopy, Zhang et al.^16^ obtained information on the Li-ion transport and chemical environment of *x*Li_2_O-MCl_*y*_ materials, while Conder et al.^17^ used synchrotron-radiation-based X-ray diffraction (XRD) to identify the absorption of polysulfide on the surface of the separator and the behavior at the electrode/electrolyte interface. Nevertheless, the above-mentioned techniques mainly focus on the Li/electrolyte interface instead of providing more details about the inner morphology and the crystal orientation evolution of the electrolyte during cycling. When tuning the incident angle of the X-ray beam to grazing-incidence conditions (<1°), methods such as grazing-incidence small-angle X-ray scattering (GISAXS) and grazing-incidence wide-angle X-ray scattering (GIWAXS) can provide more valuable information from a larger area due to the large footprint on the buried morphology, structure, and orientation of the bulk film. With this in mind, we utilized simultaneous *operando* GISAXS and GIWAXS measurements to Li||Cu cells together with cyclic voltammetry (CV) electrochemical testing to probe the morphology and structure evolution within the electrolyte. The sample position system with specific detector pixel size and sample-to-detector distance (SDD) enable the realization of spatial resolution. The high brilliance synchrotron X-ray source and fast readout time of the detector support the temporal resolution. To exclude any additive effects on the lithium plating/stripping process, a basic PCE consisting of the PEO matrix, LiTFSI salt, and Al_2_O_3_ inorganic filler without further modification is used in a Li||Cu cell. Through a comprehensive approach that incorporates CV sweeps, we investigate the mechanisms underlying the crystalline/crystallite orientation and morphology evolution of electrolytes and their interaction with electrochemical reactions on the nanoscale. Our research focuses on elucidating the properties and behavior of the electrolyte with real-time GIWAXS/GISAXS monitoring to enhance the knowledge in composite electrolyte research. Our investigation not only sheds light on these fundamental mechanisms but also opens up new paths for the designing of composite electrolytes tailored for next-generation LMBs.

## 2 Results and Discussion

**Figure 1:**
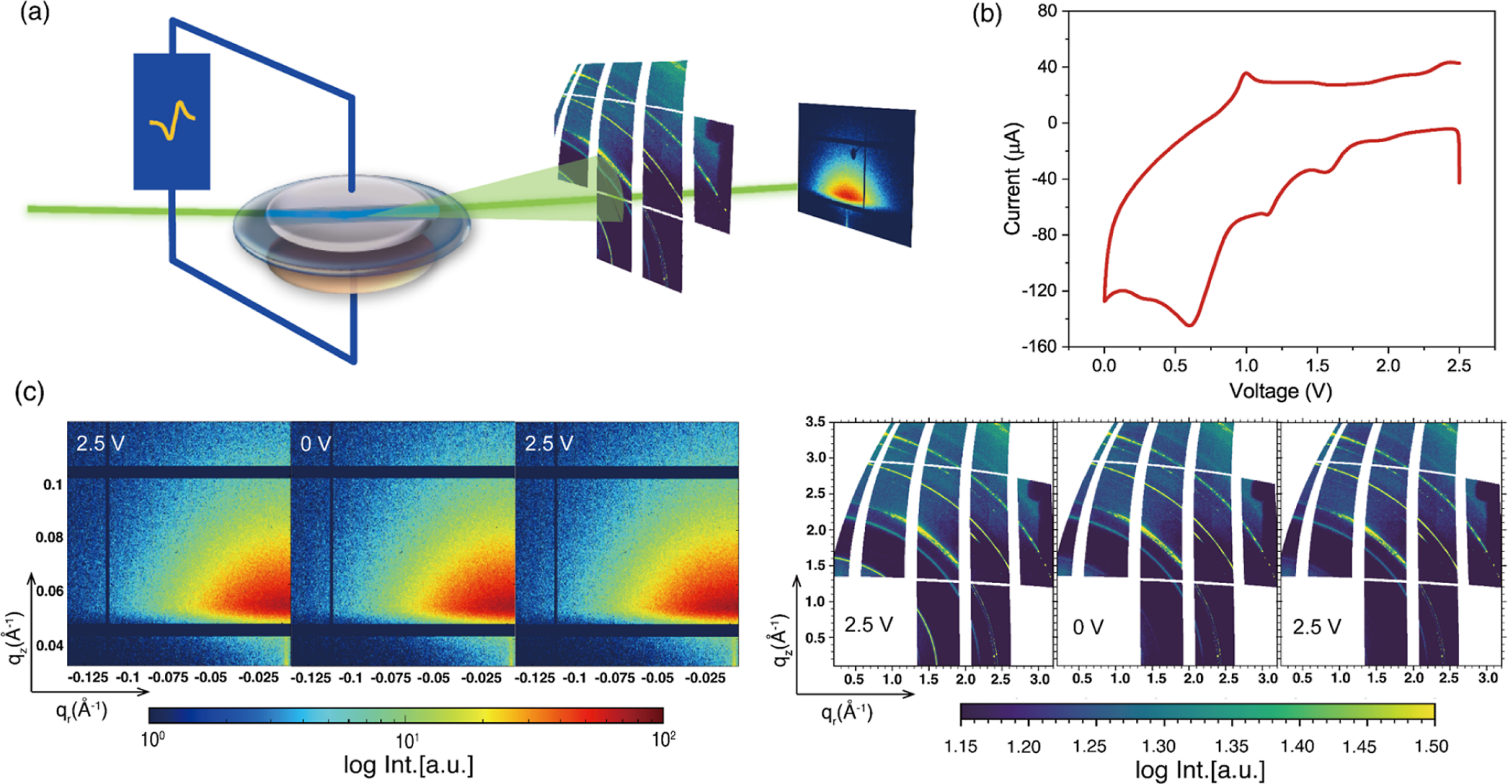
Overview of the *operando* experiment. (a) Sketch of the *operando* study of a Li|PEO composite electrolyte|Cu cell by synchrotron-radiation-based simultaneous GISAXS and GIWAXS measurements, (b) recorded CV profile of the above-mentioned cell during the GISAXS/GIWAXS measurement, and (c) selected *operando* 2D GISAXS and 2D GIWAXS data.

Figure 1a shows a sketch of the *operando* setup, where the electrochemical cell is connected to the CV test and the X-ray beam directly impinges on the electrolyte by a cut slit in the metallic lithium anode. Figure S1 provides a real photo of this setup installed at the beamline, where the GISAXS and GIWAXS detectors are placed in a way to obtain both signals at the same time. Figure 1b shows the CV curve recorded during the GISAXS and GIWAXS measurements. More details about the CV tests can be found in the Experimental Section. In the cathodic sweep, the broad and weak peak observed at around 1.95 V belongs to the initial formation of Li_*x*_CuO. With sweeping, the peaks at around 1.57 and 1.15 V correspond to the reduction of PEO-Li^+^ where the coordination bond is degraded under voltage,^18^ and the decomposition of TFSI^–^^19^ happens for the SEI layer formation, respectively. The peaks at around 0.62 V and in the 0.03–0.25 V region correspond to the Li^+^ deposition process. In the oxidation process, the peak at 0.88 V is considered as the Li^+^ stripping process, corresponding to the Li^+^ depositing process at around 0.62 V of the reduction process. The two decomposition peaks are not observed from the oxidation process due to an irreversible electrochemical reaction. Two broad peaks at 2.07 and 2.41 V can be attributed to the oxidation of Cu to Cu_2_O and CuO. In the following sections, we select four representative CV peaks to analyze. Selected typical 2D GISAXS and 2D GIWAXS data are shown in Figure 1c at the initial, middle, and final operation voltage obtained during the *operando* measurement. We note that due to the simultaneous GISAXS/GIWAXS measurement setup, the 2D GIWAXS data have a missing square (for the GISAXS detector) at lower q values (Figure 1c). To assess the consistency of our operando setup and coin cell configuration, a CV sweep is conducted on a Li||Cu coin cell (Figure S2). Despite the different currents, the electrochemical reactions remain consistent with those depicted in Figure 1b, demonstrating the effectiveness of our operando setup.

### 2.1 Crystalline Structure Evolution

**Figure 2:**
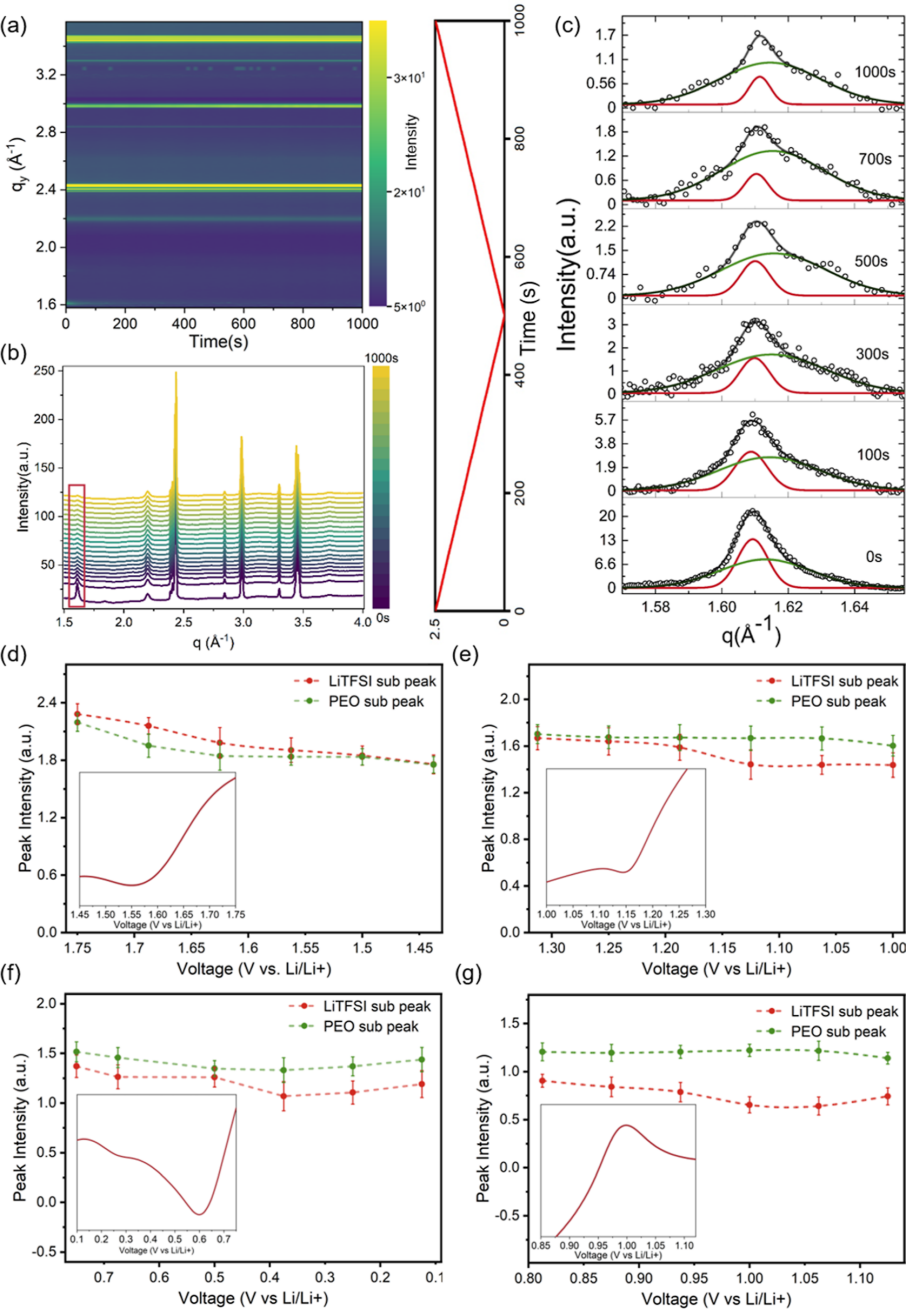
Crystalline structure evolution of the PEO composite electrolyte. (a) Temporal 2D contour plot and (b) pseudo-XRD curves acquired from cuts of the 2D GIWAXS data, with a time–voltage function on the right. (c) Zoom-in of the peak at q = 1.6 Å^–1^ probed at different CV operation times. This peak originates from the PEO-Li coordination peak. The data (symbols) are shown together with their fits (lines), which have contributions from the LiTFSI subpeak (red) and the PEO subpeak (green). (d–g) Intensity of the LiTFSI subpeak and the PEO subpeak as a function of the CV voltage sweep with the inserted corresponding electrochemical reaction peak in the CV curve.

The evolution of the crystal structure of the PCE is investigated by *operando* GIWAXS during electrochemical CV measurements. For each scattering image, the data acquisition time is 1 s, and the time interval between two images is 12.5 s. The reshaped 2D GIWAXS data from 2.5 to 0 V and from 0 to 2.5 V are shown in Figures S3 and S4, respectively. The Bragg rings in the 2D GIWAXS data originate from the crystalline part in the PCE. From the 2D GIWAXS data, we observe a diminishing intensity ring located at 1.6 Å^–1^ (Figure S3) in the reduction process of the CV (2.5 V–0 V). To visualize the changes, a contour plot of the cake cuts of 2D GIWAXS data in the range of (0°, 90°) in the χ direction is presented in Figure 2a. Figure S5a,b gives an illustration of the cake cut direction and the cake cut results, respectively. The enlarged plots at 1.6 Å^–1^ are shown in Figure S6a. Pseudo-XRD data is extracted from the 2D GIWAXS data as seen in Figures 2b and S6b.^20,21^ By comparison with the literature, it is widely recognized that the peaks observed at q = 1.6 Å^–1^ and q = 2.2 Å^–1^ correspond to the coordination peak of the PEO-LiTFSI coordination group.^22−24^ For further analysis, we designate the peak at q = 1.6 Å^–1^ as our domain peak. The intensity decrease is attributed to the reduction process of the PEO-Li^+^ group at 1.57 V in CV (Figure 1b) where the coordination bond unfolds to release free Li^+^ for further deposition. Notably, this commonly used coordination peak needs two subpeaks for modeling the intensity peak at 1.6 Å^–1^, indicating the existence of both, the PEO subpeak (green line) and the LiTFSI subpeak (red line), as shown in Figure 2c. In addition, with the applied voltage changing, also the intensity of both subpeaks changes. Initially, the coordination peak originates from LiTFSI because less EO chains can participate in the coordination with LiTFSI due to the semicrystalline nature of PEO.^25,26^ During the voltage sweep, the PEO contribution to the coordination peak becomes dominant. To quantitatively analyze the evolution of both subpeaks, we model the pseudo-XRD data and extract the intensity, full width at half-maximum (FWHM), and peak position shifts of the two subpeaks during the CV test, as shown in Figures S7–S9, respectively. The electrochemical process modifies the coordination interaction between PEO and LiTFSI, as evidenced by the scattering results. To visualize the relationship of the subpeaks’ intensity evolution and reduction/oxidation peaks observed in the CV curve, as shown in Figure 2d–g, we select four representative CV peaks and follow the intensity of both peaks across different voltage intervals. As shown in Figure 2d, when the voltage decreases from 1.75 to 1.45 V, the peak is mainly caused by LiTFSI, and the intensity of both PEO and LiTFSI subpeaks decreases, indicating that both phases joined the first electrochemical reaction in the PCE, namely, the PEO-Li^+^ reduction reaction, under the driving force of the applied potential. When the voltage is further decreased from 1.3 to 1.0 V (Figure 2e), the TFSI^–^ reduction reaction and the SEI formation start as indicated by the decreased intensity of the LiTFSI subpeak. In contrast to LiTFSI, the intensity of the PEO subpeak becomes dominant in the coordination peak and its intensity remains unchanged. In the lithiation process, the intensity of the PEO subpeak shows a slight decline followed by an increase, while the subpeak of LiTFSI shows the opposite trend within the voltage range from 0.7 to 0.1 V of the Li plating process (Figure 2f). This phenomenon can be attributed to the diffusion of Li ions from the Li metal side to the Cu side. When the last clear Li stripping CV peak is reached, as shown in Figure 2g, the intensity of the LiTFSI subpeak decreases from 0.8 to 0.6, while the intensity of the PEO subpeak increases. This trend suggests that Li^+^ from the Cu side diffused through the PEO segment to the Li side. However, the intensity of the coordination peak does not increase again, meaning that the free Li ions cannot be incorporated again within the PEO chains to form the PEO–Li^+^ bond. Thus, throughout the entire CV sweep, the evolution of LiTFSI and PEO subpeaks aligns consistently with the electrochemical reactions. Apart from the intensity change, the increased fwhm is attributed to the lower crystallinity of PEO in the coordination peak induced by the voltage stimulus (Figure S8). These observations collectively suggest the presence of a more amorphous phase within the PCE, which facilitates the Li^+^ transport process. Moreover, the decreased fwhm of the LiTFSI subpeak originates from residual LiTFSI, which cannot coordinate with the PEO chains at room temperature. The shrinkage of the lattice distance, inferred from the peak position in Figure S9, indicates the existence of stress within the electrolyte, as well as the irreversibility of both components following the processes of Li plating and stripping, leading to adverse effects on the electrochemical performance of the cell.

**Figure 3:**
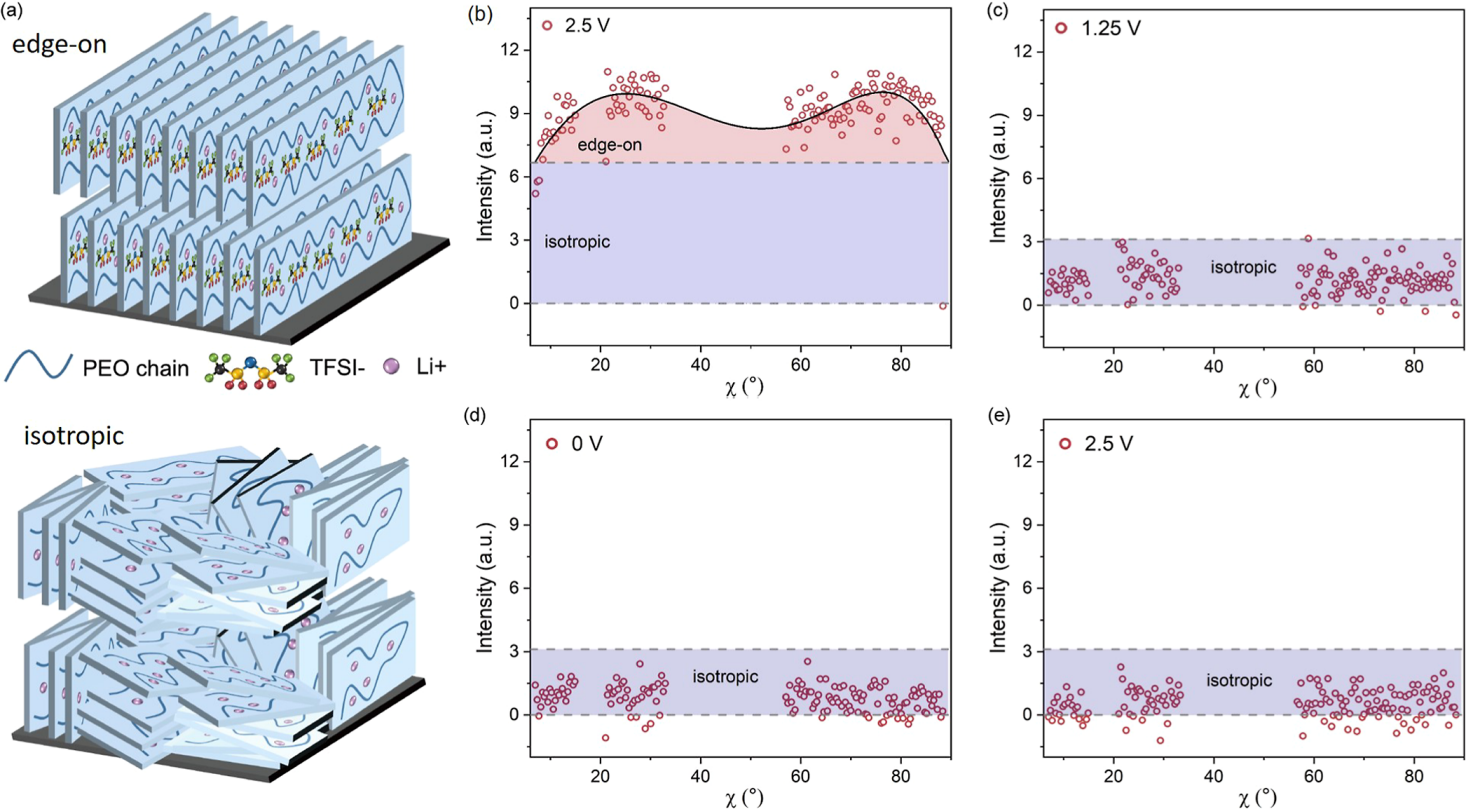
Crystalline orientation evolution of the PEO composite electrolyte. (a) Illustration of the orientation changes from edge-on orientation to an isotropic arrangement. Azimuth tube cuts (points) with fits (lines) of 2D GIWAXS data at q = 1.6 Å^–1^ during the CV sweep: (b) 2.5 V (0 s), (c) 1.25 V (250 s), (d) 0 V (500 s), and (e) 2.5 V (1000 s).

In contrast to powder XRD, GIWAXS offers insights into the preferred crystal orientations. Therefore, in addition to the cake cuts, azimuthal tube cuts are performed at q = 1.6 Å^–1^ to obtain the preferred crystallite orientation information. An illustration of the tube cuts is given in Figure S5a,c. The schematic representation of edge-on and isotropically oriented crystallites is given in Figure 3a. In an edge-on oriented structure, the crystallites are in a perpendicular direction to the film, while in an isotropic oriented structure, the arrangement of crystallites is disordered without any preferred orientation.^27^ The PEO-Li demonstrates an edge-on orientation at approximately χ = 20 and 80°, as shown in Figure 3b, aligning with crystals oriented in 20 and 80° directions to the substrate before the voltage sweep. The crystallite orientation as a function of voltage change manifests a more disordered status in the PCE, as shown with the tube cuts at different voltages in Figure 3c–e, transforming from an edge-on dominant structure to an isotropic structure. The isotropic orientation indicates that the PEO chains arrange in all directions, providing more Li^+^ transport pathways, which is beneficial for the Li^+^ plating and stripping processes. The observed orientation transformation is attributed to electrochemical reactions within the PCE, specifically, PEO-Li^+^ reduction and TFSI decomposition. These processes result in the breakdown of large oriented polymer chains into smaller disordered segments, facilitating subsequent Li^+^ transport. It is important to note that an X-ray radiation damage test was conducted on the PCE for 1000 s before the *operando* measurements to rule out any possible damage caused by the X-ray beam (contour plot of horizontal line cuts shown in Figure S10).

### 2.2 Buried Morphology Evolution

**Figure 4:**
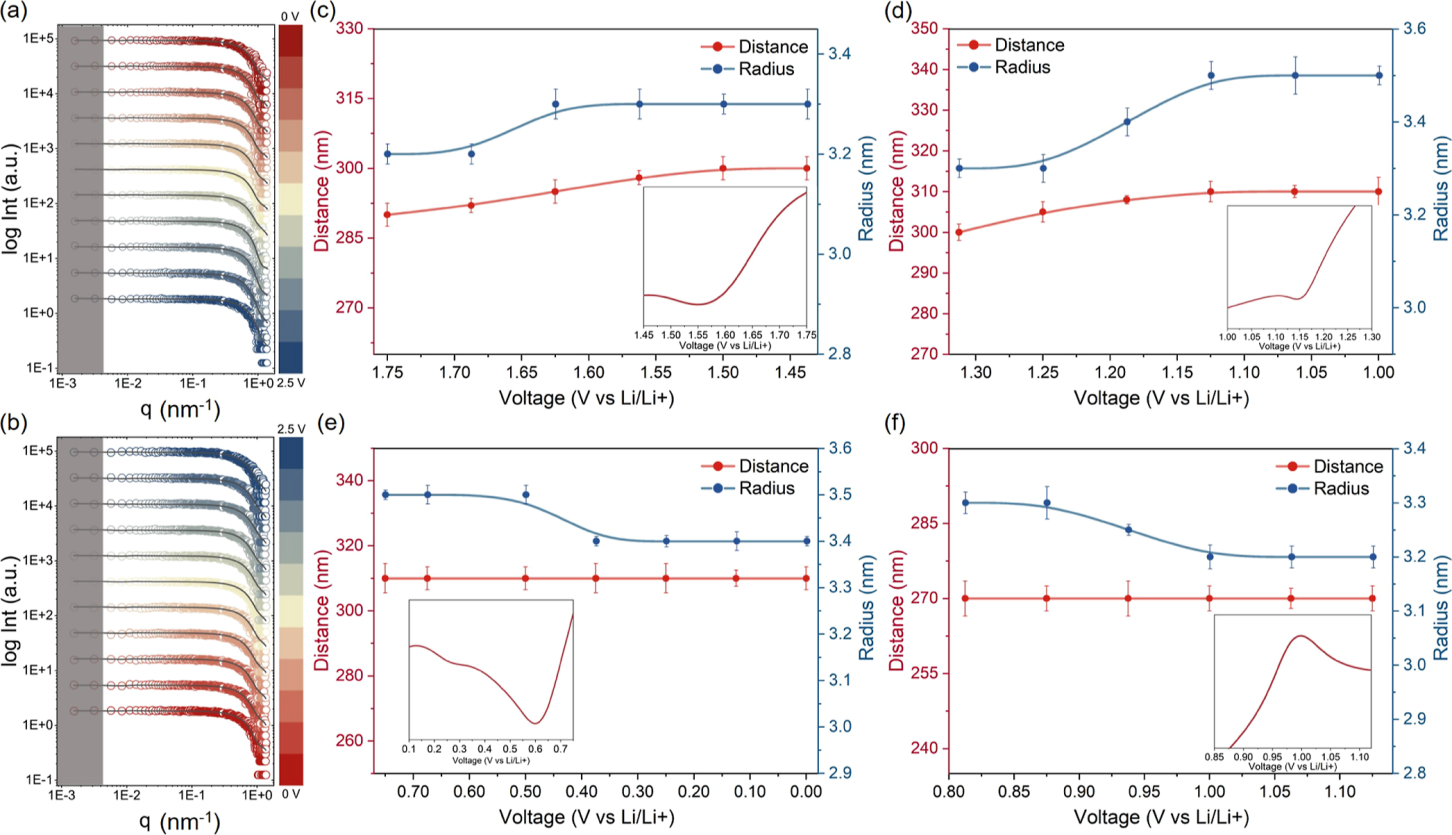
Horizontal line cuts of the 2D GISAXS data (symbols) taken at the Yoneda region of the PCE during the CV testing for (a) 2.5–0.0 V and (b) 0.0–2.5 V. The best fits are shown with lines. The gray region represents the resolution limit and the curves are shifted along the *Y*-axis for data readability. (c–f) Extracted fit parameters in terms of radius (blue) and center-to-center distance (red) of the PEO-LiTFSI domains as a function of different applied CV voltages. The inset represents the corresponding electrochemical reaction peak in the CV curve.

In addition to the crystalline structure obtained via *operando* GIWAXS, electrochemically induced changes to the internal morphology of the PCE are detected with the simultaneously performed *operando* GISAXS measurements. The grazing-incidence angle of 0.4° is chosen to be above the critical angle of each component in the PCE, which ensures that the inner morphology of this material is probed.^28,29^ The selected 2D GISAXS data at different operation times are given in Figure S11. The horizontal line cuts are conducted at the Yoneda peak region of the PCE to quantitatively analyze the morphology evolution, and Figure S12 illustrates the horizontal line cut direction. The line cut data is modeled in the framework of the distorted wave Born approximation (DWBA).^30,31^Figure 4a,b shows the horizontal line cuts with best fits in a series of voltage changes. From these fits, the form factor (radius) and structure factor (center-to-center distance) are extracted as shown in Figure S13. During the negative CV sweep (from 2.5 to 0.0 V), the radius of the PEO-LiTFSI domains increases from 3.0 to 3.4 nm, and the related center-to-center distances show the same trend by increasing from 240 to 310 nm. In the second step of the CV (from 0.0 to 2.5 V), the radius and the center-to-center distance show a reversed tendency and change into a smaller radius and shorter distance. Notably, the changes in radius and distance during the CV test are in accordance with the observed peaks shown in Figure 1b. Similarly, the radius and center-to-center distance changes at various voltage intervals are extracted along with the corresponding reduction–oxidation CV peaks, as shown in Figure 4c–f. When the voltage sweeps from 1.75 to 1.45 V (Figure 4c), the distance increases from 290 to 300 nm, consistent with the PEO reduction reaction. The increase in distance and radius implies that Li ions escape from the PEO-LiTFSI coordination group for further reaction. With continuous voltage decrease to 1.0 V (Figure 4d), the distance expands from 300 to 310 nm as well as the radius increases to 3.5 nm, indicating the reduction and decomposition of TFSI^–^ anions and a SEI formation.^32,33^ In the underpotential deposition of Li at 0.62 V and in the voltage range of 0.25–0.03 V according to the CV curve (depicted in Figure 4e), a decrease in the radius of PEO domains is observed. However, no concurrent change in the distance between two neighboring domains is seen. This finding suggests that the Li^+^ diffusion is occurring through the PEO chain. As a consequence of a number of free Li^+^ transporting to the Cu side, a continuous formation and breaking of the Li–O bond with each ether oxygen atom from PEO domains occurs. This process contributes to the diminishing radius of the PEO domains. Furthermore, it is noteworthy that ion diffusion does not induce a movement in the PEO chain, thereby maintaining the unchanged distances between domains. With a continuous voltage increase (Figure 4f), the PEO domain radius continues to decrease while the distance remains constant, indicating that Li^+^ can be transported into the PCE through the polymer chain segments but cannot reform the PEO-Li^+^ coordination groups.^34^ We tabulate the observed crystalline structure and buried morphology variations across various sweep voltages together with the corresponding electrochemical reactions.

These comprehensive findings are detailed in Table S1, providing a summary of the relationship between sweep voltage and the associated structural and morphological modifications, as well as the electrochemical responses.

### 2.3 Electrochemical Performance

Apart from the CV, which has been collected in the *operando* measurement, the electrochemical and cycling performances are presented in Figure S14 to prove the effectiveness of the PCE in a coin cell framework. As seen from the electrochemical performance shown in Figure S14a, the PCE exhibits a good electrochemical stability with an ionic conductivity of 1.34 × 10^–4^ S cm^–1^ and a broadened electrochemical window up to 4.8 V (Figure S14b) in spite of the oxidation of the −OH group above 4 V (vs Li/Li^+^).^35,36^ These results show that the dissociation of TFSI^–^ with PEO chains sets off more free Li^+^ ions to increase conductivity and protects the PEO chain from oxidation.^37,38^ Li symmetrical cells are assembled to further estimate the stability of the PCE to the Li anode (Figure S14c). The PCE presents a high Li^+^ transference number of 0.38 and a minor interfacial resistance before and after polarization. Accordingly, the symmetric cell containing the PCE displays an excellent long-term stability (Figure S14d) over 450 h at 0.1 mA cm^–2^, coupling with a steady overpotential of 96 mV, which is ascribed to the good ionic conductivity and Li^+^ transfer number. With the confirmed stability of the PCE to Li metal, it proves the feasibility of PCE-based LMBs in agreement with prior literature reports.^22,39−43^

## 3 Conclusions

In summary, we have investigated the evolution of the buried morphology and crystalline structure of the PCE within the Li||Cu cell under bias during a CV sweep by synchrotron-radiation-based simultaneous *operando* GISAXS and GIWAXS measurements. The electrochemical reactions within the PCE are strongly related to the observed morphology and structure changes. When the PCE undergoes the PEO reduction process, the PEO-LiTFSI domain radius and the center-to-center distance increase, while the intensity of the PEO-Li^+^ coordination Bragg peak decreases due to the disconnection of the PEO-Li^+^ coordination bonds. Moreover, the increase in the PEO-LiTFSI domain radius at a bias of approximately 1.15 V and the decreased intensity of the LiTFSI Bragg peak are observed as a result of the TFSI^–^ decomposition reaction and SEI formation. During the Li^+^ stripping process at 0.88 V, the crystalline structure remains unchanged, whereas the inner morphology changes are ongoing, implying the Li ion diffusion but not building of the PEO-Li^+^ bonds. Moreover, the crystallite orientation of the PCE changes from an edge-on domain to an isotropic domain type, meaning that a more disordered and amorphous structure of the PCE is generated under the applied voltage, which is beneficial for ion transport through polymer chains. In addition to this, the moderate electrochemical properties and battery performance also demonstrate the feasibility of LMBs with the probed PCE acting as a solid electrolyte. Thus, our work develops pioneering *operando* measurements using grazing-incidence X-ray scattering techniques and contributes to an understanding of the lithium-ion plating/stripping process by probing morphology and structure changes in real time, which can provide a guideline for development of high-CE and high-conductivity LMBs.

## 4 Experimental Section

### 4.1 Materials

PEO (average Mv = 600,000 g/mol), Al_2_O_3_ (<50 nm nanopowder), lithium bis(trifluoromethanesulfonyl)imide (LiTFSI, 99.95% trace metals basis), poly(vinylidene fluoride) (PVDF), acetonitrile (ACN, electronic grade, 99.999% trace metals basis), and *N*-methyl-2-pyrrolidone (NMP, anhydrous, 99.5%) were purchased from Sigma-Aldrich. Conductive carbon black (Super P) and LiNi_0.8_Co_0.1_Mn_0.1_O_2_ (NCM811) powder were purchased from NEWARE TECHNOLOGY LIMITED. All chemicals were used as received without further treatment.

### 4.2 Preparation of a PEO Composite Electrolyte

A PEO composite electrolyte was fabricated by the solution casting method. PEO and LiTFSI were dissolved in the ACN with a ratio of EO/Li = 14. Afterward, 10 wt % of Al_2_O_3_ nanopowder was added, and the above-mentioned solution was constantly stirred at 50 °C for 24 h. Then the solution was poured on a Teflon plate, followed by solution evaporation in an argon atmosphere for 12 h to obtain a homogeneous free-standing electrolyte.

### 4.3 Assembly of All-Solid-State LMBs

CR2032 coin cells were assembled by sandwiching the electrolyte embedded in two stainless discs, lithium||stainless disc, and two lithium chips in an argon-filled glovebox (O_2_, H_2_O < 0.1 ppm) to avoid any contamination.

### 4.4 Electrochemical Characterization

The electrochemical measurements of the PEO composite electrolyte were carried out on a VMP300 Biologic electrochemical workstation at a working temperature of 55 °C. For ionic conductivity, the frequency range was from 1 MHz to 0.1 Hz with an AC voltage amplitude of 10 mV and was applied to a symmetric cell with two stainless steel electrodes. The ionic conductivity σ was calculated following a previously reported approach, according to the following equation.^44^
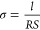
where *l* refers to the thickness of the electrolyte, *R* refers to the resistance, and *S* refers to the electrode area.

The Li^+^ transfer number was calculated by the potentiostatic polarization method with a 10 mV constant voltage on a lithium symmetric cell according to a previously reported method.^45^ The electrochemical impedance spectroscopy measurement was performed on the cell before and after polarization. The Li^+^ migration number *t*_Li^+^_ is then calculated using the following equation
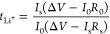
where *I*_0_ and *I*_s_ represent the initial and steady-state current after polarization and *R*_0_ and *R*_s_ represent the interfacial resistance before and after polarization, respectively, and Δ*V* is the polarization voltage, which in our case is 10 mV.

The linear sweep voltammetry test was carried out on the PEO composite electrolyte by placing the electrolyte between a metallic lithium anode and a stainless steel chip and was measured with a sweep rate of 1 mV s^–1^.

### 4.5 Galvanostatic Charge/Discharge Cycling

For the charge/discharge process of the lithium symmetric cell, the tests were conducted at a current density of 0.1 mA cm^–2^ for 1 h per cycle, and the working temperature is 55 °C.

### 4.6 Operando Experiment

GISAXS and GIWAXS were performed at the beamline P03 (MiNaXS beamline) of the PETRA III storage ring, Deutsches Elektronen-Synchrotron (DESY, Hamburg, Germany),^46^ with a beam energy of 12.9 keV to avoid absorption edges and radiation damage. The PILATUS 2M detector (Dectris, pixel size 172 μm × 172 μm) and LAMBDA 9M detector (X-Spectrum, pixel size 55 μm × 55 μm) were located at 4090 and 293.15 mm SDDs, respectively, to capture the GISAXS and GIWAXS signals simultaneously. The incident angle was chosen above the critical angles of the involved materials and set to 0.4° to balance the penetration depth and background. The background was further minimized by utilizing a high X-ray beam energy.

### 4.7 Operando Setup at P03 MiNaXS Beamline

The operando setup consisted of a homemade cell with two attached Kapton windows on both the front and back sides and a VMP150 Biologic electrochemical workstation. Figure S1 shows a photograph of the complete setup at beamline P03. To ensure the detection of the cell in its inherent ambient conditions, two tubes were utilized. These tubes were connected to an argon gas bottle, maintaining a gas flow of approximately 1 mbar. The measurements were carried out at a temperature of 35 °C. A Li||Cu cell was used to investigate the lithium deposition process. Copper foil was used as the substrate and the electrolyte was deposited on it to realize a flat surface. In order to detect the changes within the electrolyte, a slit was made on the lithium chip and the stainless steel current collector. The cell was connected to the electrochemical workstation to record the CV curve between 2.5 and 0.0 V with a scan rate of 5 mV/s.

### 4.8 GIWAXS Correction and Treatment

The collected GIWAXS data were further processed by the Python-based heuristic tool INSIGHT.^47^ The correction of SDD induced by the chamber thermal expansion was realized by calibrating the (111) Cu Bragg reflex to 3.0103 Å^–1^ (2θ = 43.297°). Cake cuts of the 2D GIWAXS data for pseudo-XRD were done with the limits of χ = [0, 90°] and q = [1.49, 4.01] Å^–1^. Azimuth tube cuts were conducted with the limits of χ = [0, 90°] and q = [1.55, 1.65] Å^–1^, and the background subtraction was done by INSIGHT.

### 4.9 GISAXS Data Treatment and Crystal Size Distribution

The theoretical critical angles for LiTFSI (0.1078°), PEO (0.1021°), and Al_2_O_3_ (0.1025°) at the above-mentioned X-ray energy were calculated by the SLD Python package. Analysis of the GISAXS data was performed via DPDAK software.^48^ The Yoneda peak position was identified via vertical line cuts of the 2D GISAXS data. Horizontal line cuts were taken in the Yoneda region to obtain the structural information. The horizontal line cut data was modeled in the framework of DWBA using the local monodisperse approximation.^29,49^ Based on this, the diffuse scattering intensity ***I***(***q⃗***) was modeled based on the following equation
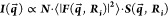
where ***N*** represents the total number of scattering objects, ***F*(*q⃗*)** the form factor, and ***S*(*q⃗*)** the structure factor.

In our work, we assumed that the scattering objects possessed a cylindrical shape with a radius (*R*) and a Gaussian distribution of sizes. A 1-dimensional paracrystalline model was applied to extract the distances between neighboring objects (center-to-center distance *D*).

## Supporting Information Available

Photographs of the operando setup; CV on the coin cell; selected 2D GIWAXS data; example of GIWAXS data analysis; voltage dependence of peak intensity, peak fwhm, and peak position; radiation damage test; selected 2D GISAXS data; example of GISAXS data analysis; voltage dependence of characteristic morphology parameters; and table of structure changes and electrochemical measurements of the PCE in the coin cell (PDF)
