# Supplementary material for: Operando Study Insights into Lithiation/Delithiation Processes in a Poly(ethylene oxide) Electrolyte of All-Solid-State Lithium Batteries by Grazing-Incidence X-ray Scattering
Source: ACS Appl Mater Interfaces. 2024 Jun 24;16(26):33307–15. doi: 10.1021/acsami.4c01661 (PMC11231968; doi:10.1021/acsami.4c01661)
Supplement: Supplementary file 1 — am4c01661_si_001.pdf [file am4c01661_si_001.pdf]

Supporting Information

Operando Study Insights into Lithiation/Delithiation Processes in Poly(ethylene oxide) Electrolyte of All-Solid-State Lithium Batteries by Grazing Incidence X-Ray Scattering

Yuxin Liang [1], Tianle Zheng [1], Kun Sun [1], Zhuijun Xu [1], Tianfu Guan [1], Fabian A.C. Apfelbeck [1], Pan Ding [2], Ian D. Sharp [2], Yajun Cheng [3], Matthias Schwartzkopf [4], Stephan V. Roth [4,5], Peter Müller-Buschbaum [1,]*

1. Technical University of Munich, TUM School of Natural Sciences, Department of Physics, Chair for Functional Materials, James-Franck-Str. 1, 85748 Garching, Germany

2. Walter Schottky Institute and Physics Department, Technical University of Munich, Am Coulombwall 4, 85748 Garching, Germany

3. Ningbo Institute of Materials Technology & Engineering, Chinese Academy of Sciences, 1219 Zhongguan West Rd, Ningbo, 315201, Zhejiang Province, P. R. China

4. Deutsches Elektronen-Synchrotron (DESY), Photon Science, Notkestr. 85, 22607 Hamburg, Germany

5. KTH Royal Institute of Technology, Department of Fibre and Polymer Technology, Teknikringen 56-58, SE-100 44 Stockholm, Sweden

Corresponding Author:* Peter Müller-Buschbaum muellerb@ph.tum.de

Supporting Figures

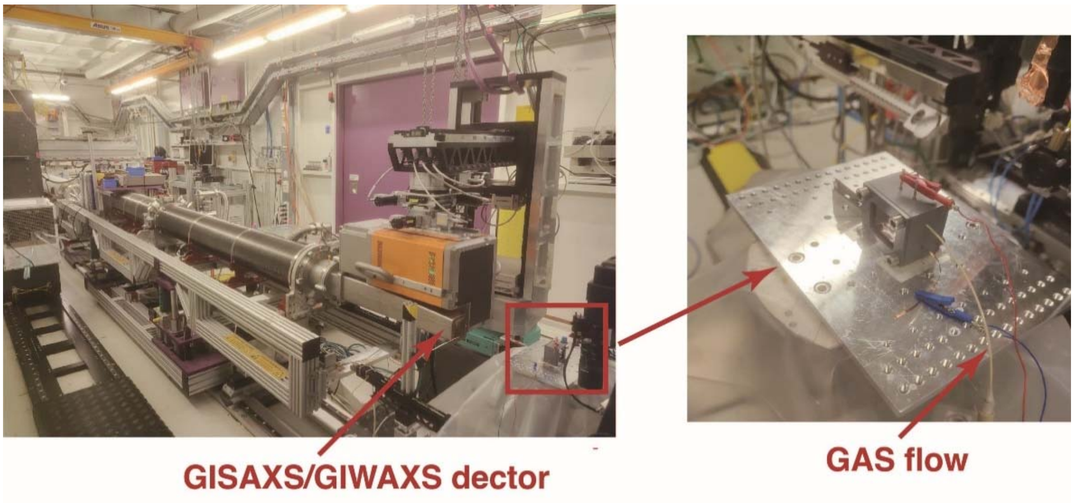

Figure S1. Photograph of the operando setup as used at the P03 beamline at DESY.

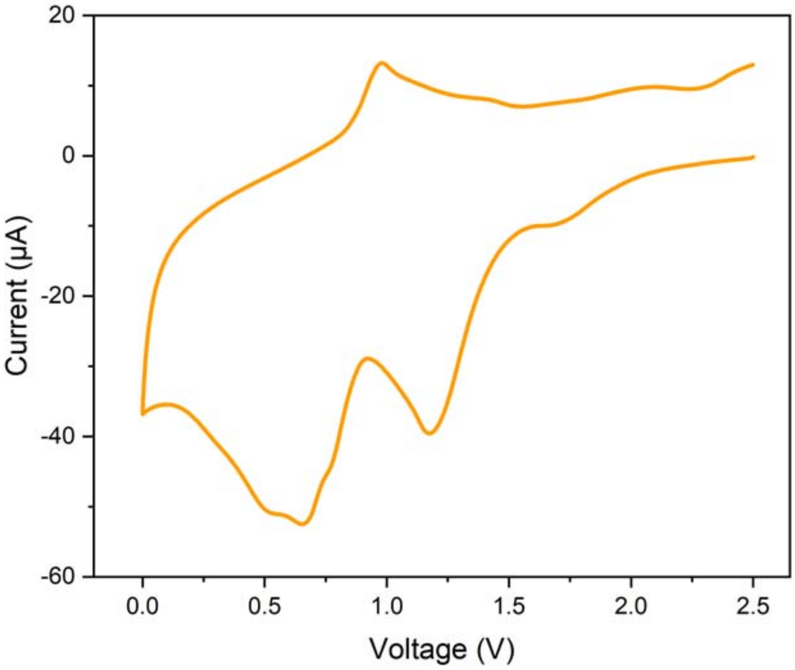

Figure S2. Cyclic voltammetry profile of Li||Cu coin cell with a scan rate of 5 mV/s.

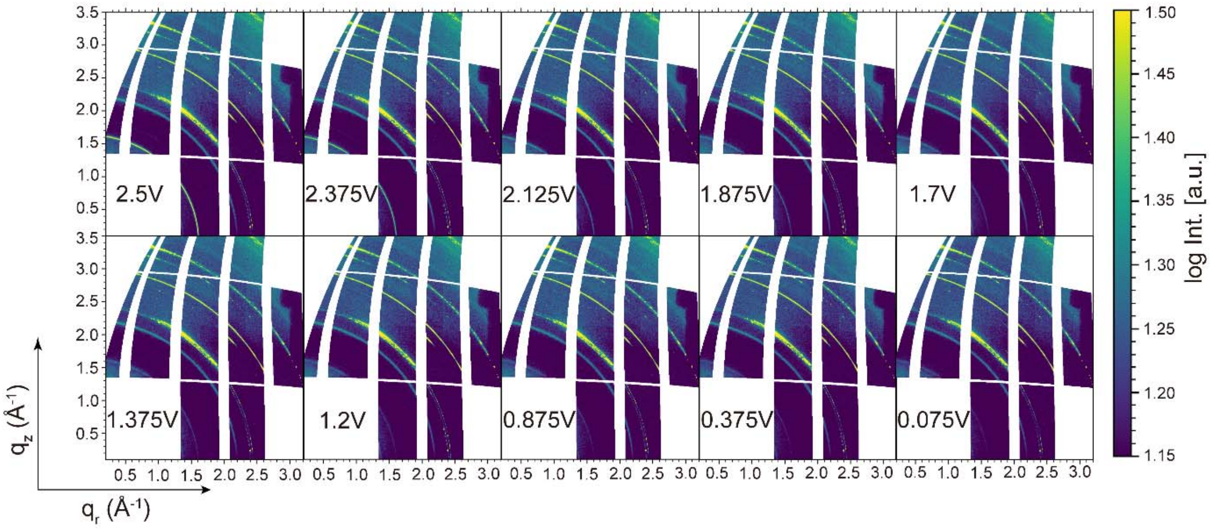

Figure S3. Selected 2D GIWAXS data of the Li| PEO composite electrolyte |Cu cell at different operational voltages from 2.5 to 0 V.

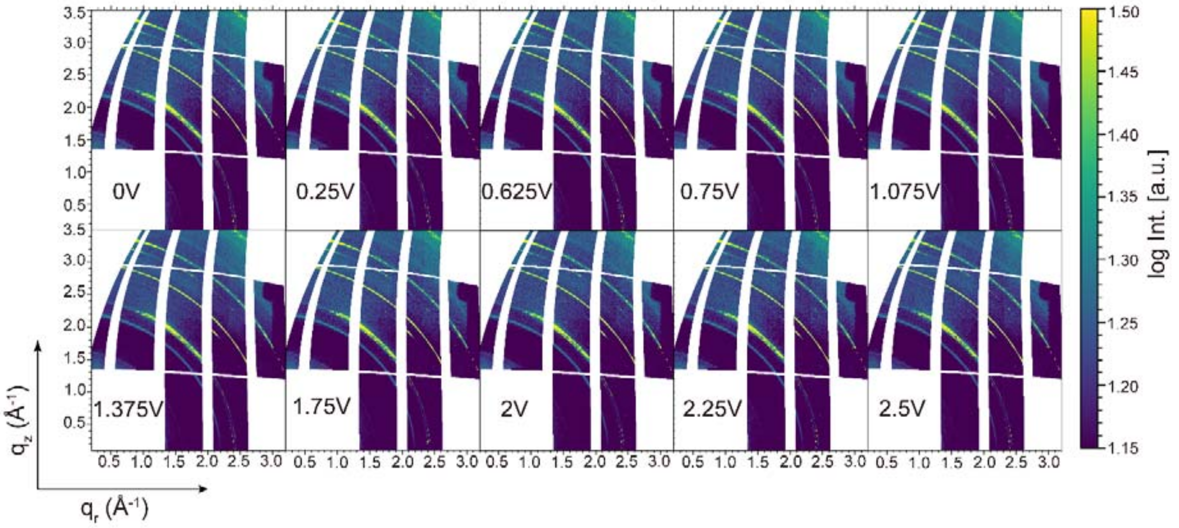

Figure S4. Selected 2D GIWAXS data of the Li| PEO composite electrolyte |Cu cell at different operational voltages from 0 to 2.5 V.

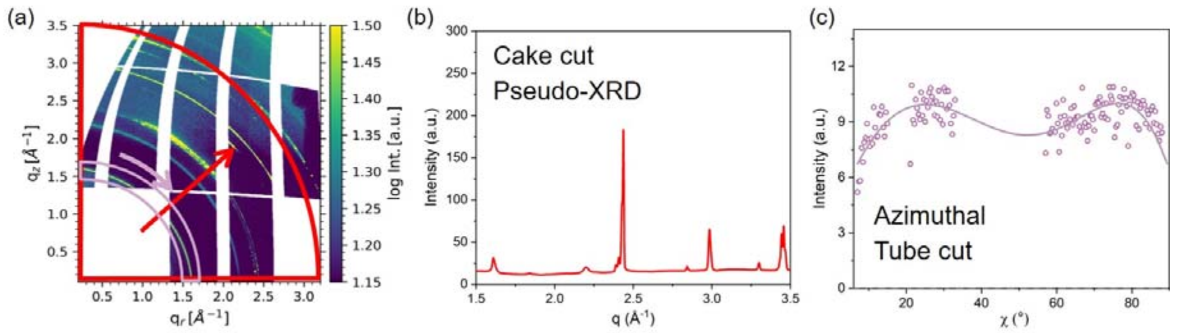

Figure S5. Example of cuts in the GIWAXS data analysis. (a) Example 2D GIWAXS data, with the red area, representing the cake cut area, and the arrow representing the cut direction; the purple ring area representing the azimuthal tube cut, and the purple arrow representing the cut direction. (b) Pseudo-XRD profile obtained from the cake cut. (c) Orientation information obtained from the azimuthal tube cut.

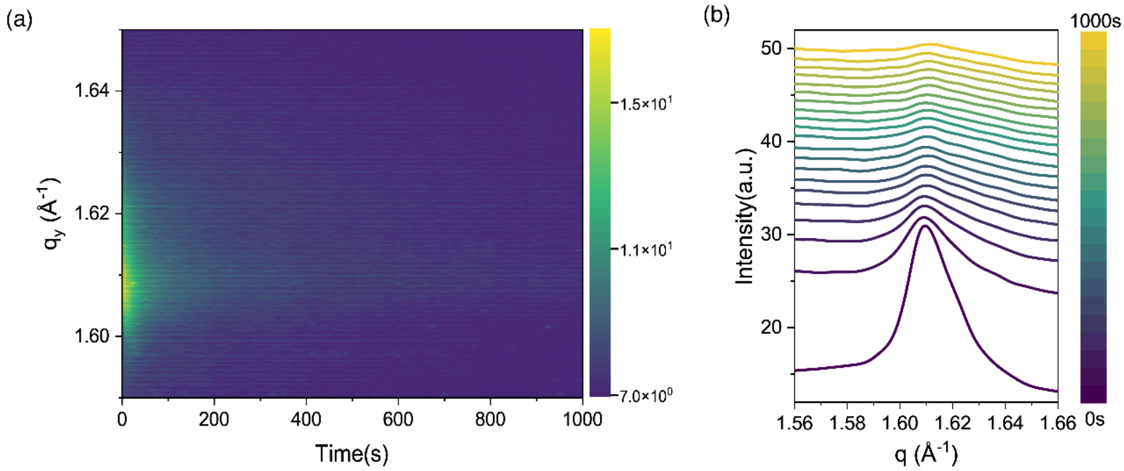

Figure S6. Enlarged contour plot (a) and pseudo-XRD cuts (b) in the q_y =1.6 Å^{-1} region.

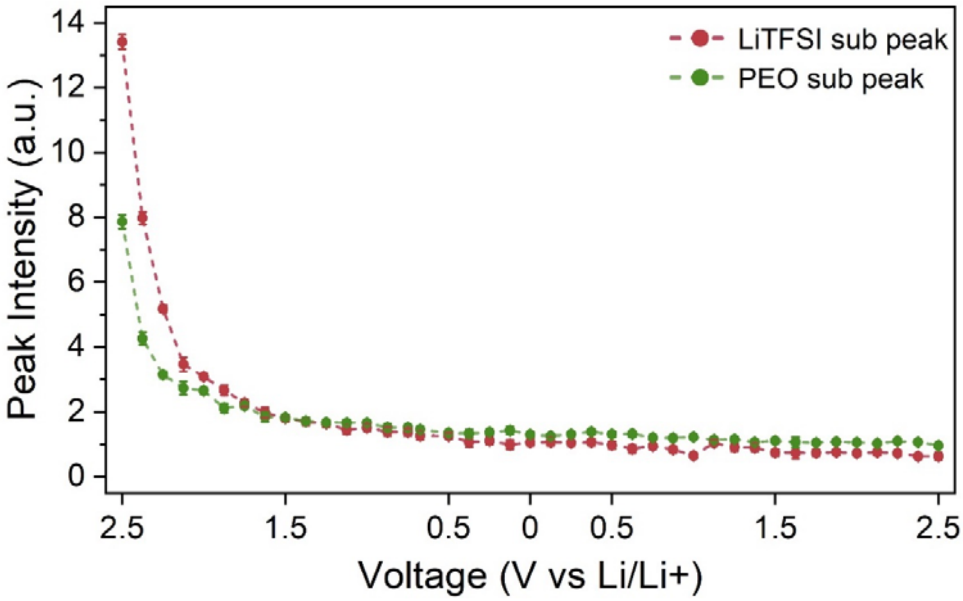

Figure S7. Peak intensity of the LiTFSI sub-peak (red) and the PEO sub-peak (green) as a function of the CV voltage sweep. Note that the sweep direction was reversed at 0 V.

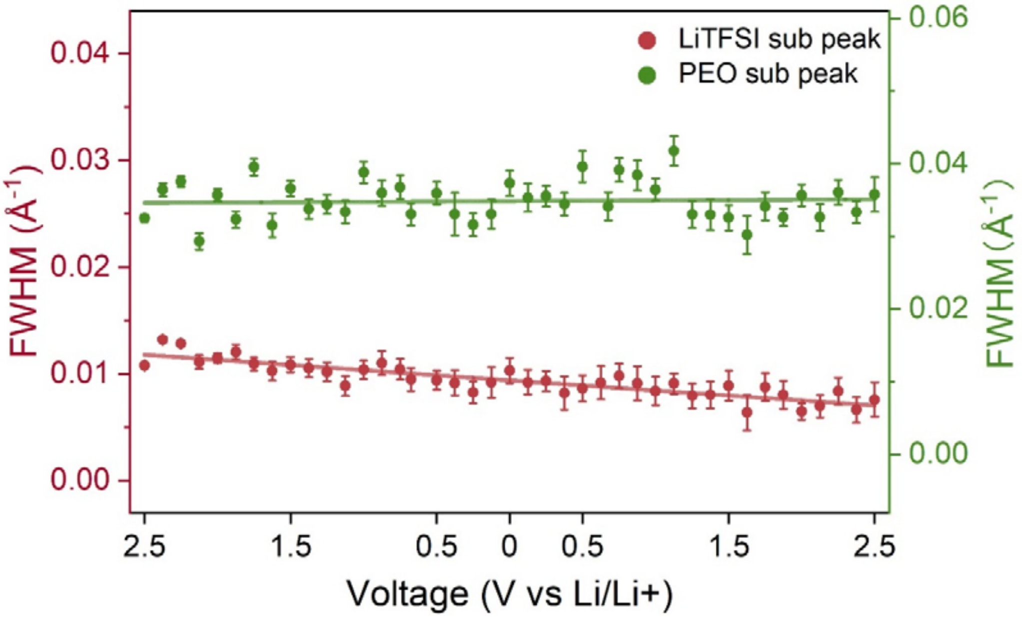

Figure S8. Full width at half maximum (FWHM) of the LiTFSI sub-peak (red) and the PEO sub-peak (green) as a function of the CV voltage sweep. Note that the sweep direction was reversed at 0 V.

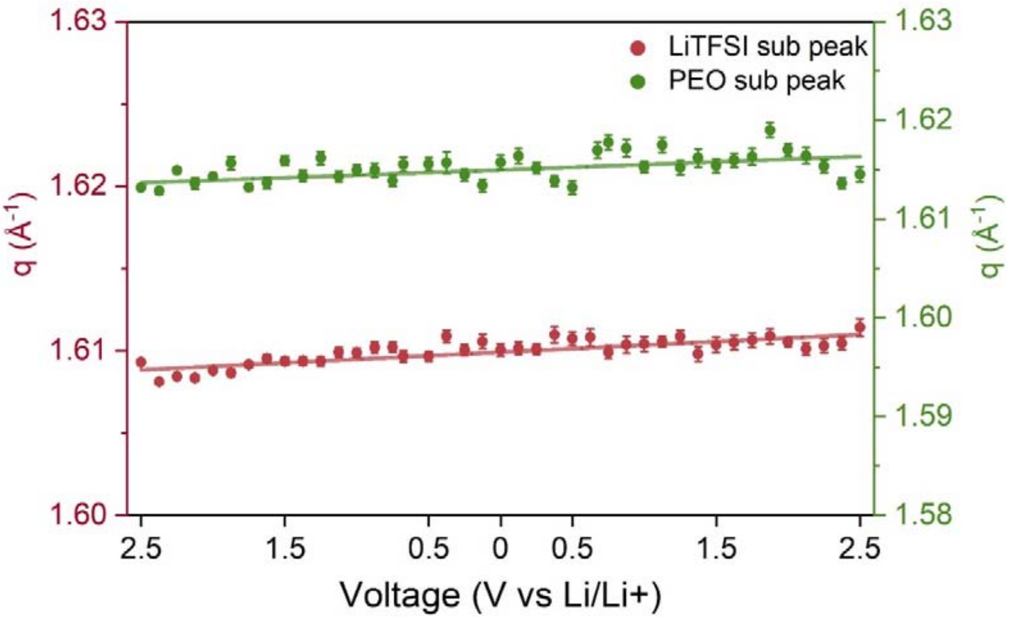

Figure S9. Peak position evolution of the LiTFSI sub-peak (red) and the PEO sub-peak (green) as a function of the CV voltage sweep. Note that the sweep direction was reversed at 0 V.

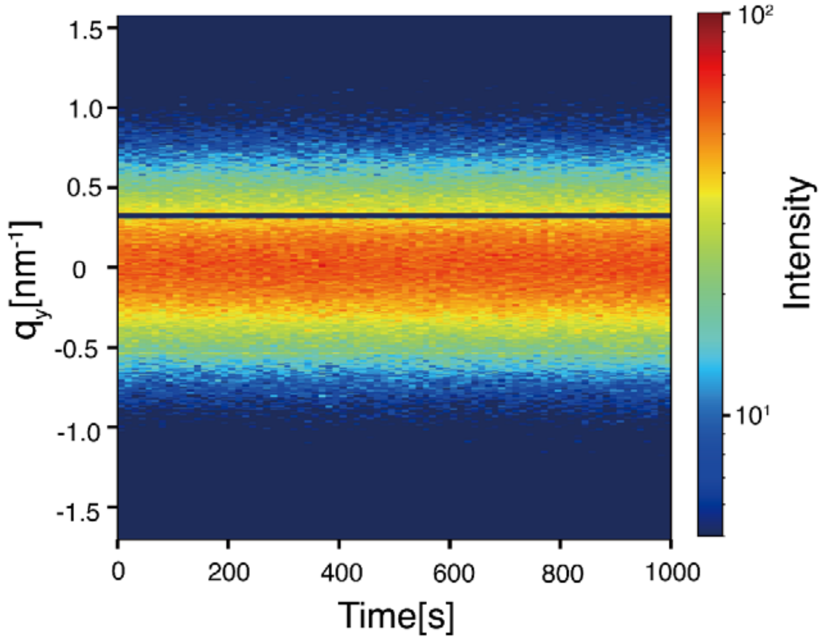

Figure S10. X-ray radiation damage test of the PEO composite electrolyte.

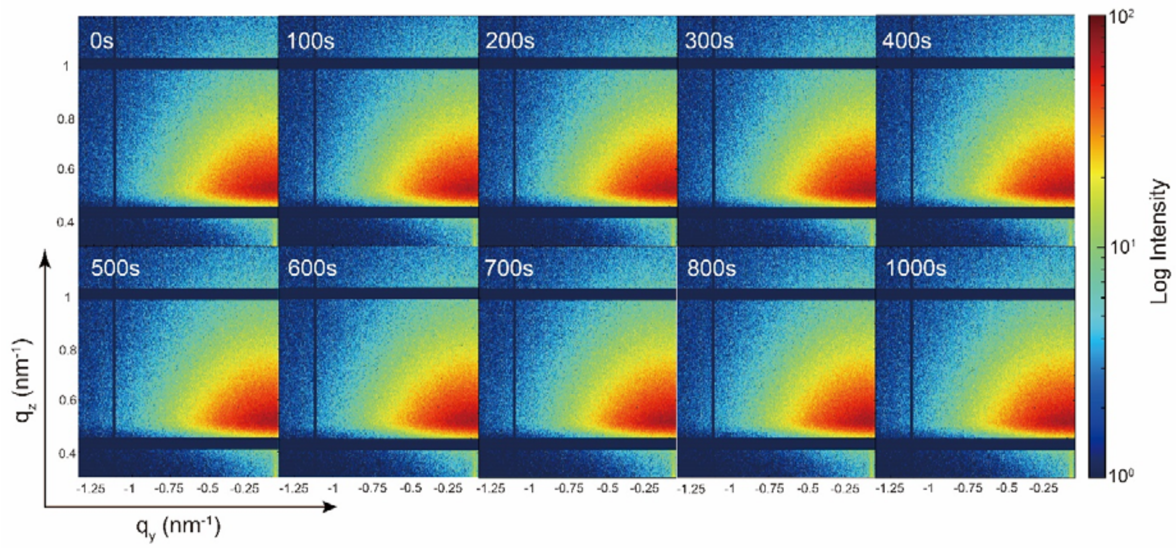

Figure S11. Selected 2D GISAXS data of Li| PEO composite electrolyte |Cu cell at different operation times.

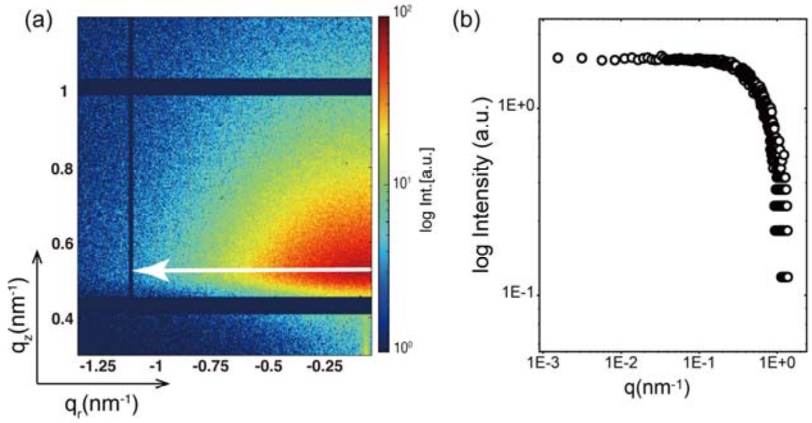

Figure S12. Example of horizontal line cut in GISAXS data analysis. (a) Example 2D GISAXS data with the white arrow representing the cut direction and (b) resulting horizontal line cut profile.

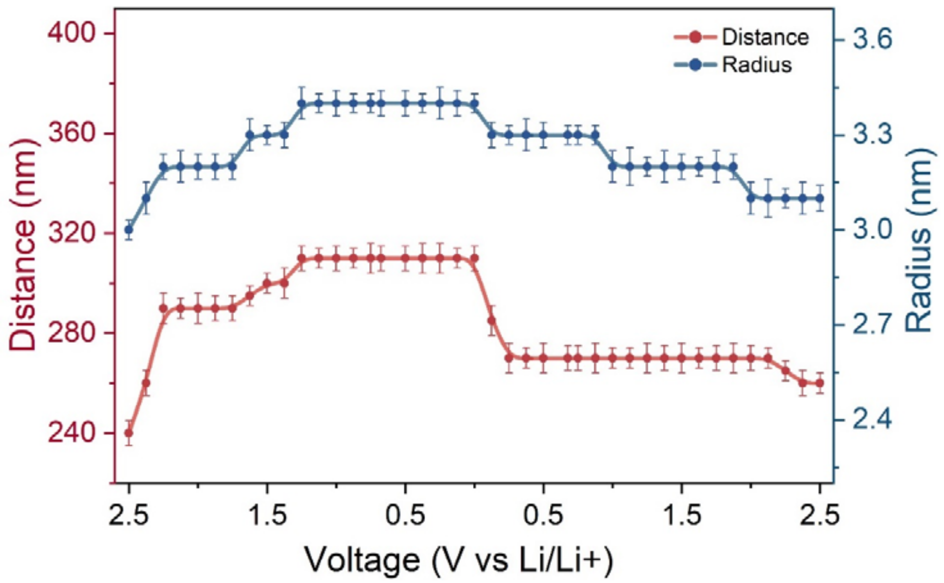

Figure S13. Extracted fit parameters in terms of radius (blue) and center-to-center distance (red) of the PEO-LiTFSI domains as a function of the applied voltage during CV sweep. Note that the sweep direction was reversed at 0 V.

Table S1. Summary of the crystalline structure and buried morphology changes at different sweep voltages and electrochemical reactions.

Voltage from CV(V vs Li/Li+)	Electrochemical reactions	Crystalline structure	Morphology
1.57	EO-Li reduction	Intensity of both sub-peaks decreased then increased, and orientation became more isotropic.	EO-Li domain shows larger radius, and distance between domains increased.
1.15	$TFSI^-$ decomposition	Only LiTFSI sub-peak shows decreased intensity.	Radius and distance of domains continuously increasing.
0.62	Li^+ plating	Intensity of both sub-peaks decreased then increased.	Li^+ transport process, only the domain radius changes.
0.88	Li^+ stripping	Reversed tendency of Li^+ plating process.	Li^+ transport process, only the domain radius changes.

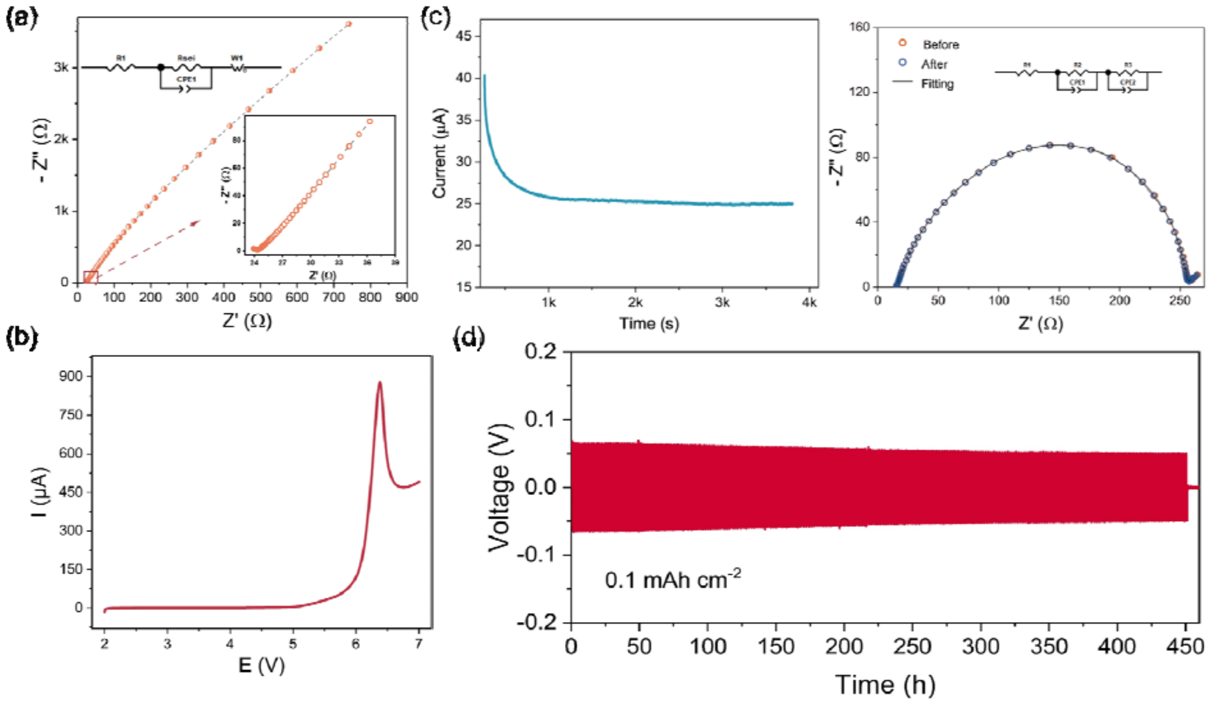

Figure S14. Electrochemical measurements on PCE in coin cell configuration. (a) Impendence spectra for calculation of the ionic conductivity. (b) Linear sweep voltammetry curve of the PEO composite electrolyte for the electrochemical window. (c) Steady current as a function of time recorded during 10 mV polarization on the Li symmetric coin cell and EIS plots before and after the polarization. (d) Voltage-time profile of the Li symmetric coin cell measured at a current density of 0.1 mA cm^{-2}.
